# Comparative analysis of osteointegration in photostimulated dental implants: 650-976 nm diode lasers versus growth factors

**DOI:** 10.25122/jml-2023-0281

**Published:** 2024-08

**Authors:** Zahraa Abdulrazaq Alameeri, Hussein Ali Jawad

**Affiliations:** 1 Institute of Laser for Postgraduate Studies, University of Baghdad, Baghdad, Iraq

**Keywords:** biostimulation, diode lasers, implant stability quotient, photobiomodulation, platelets-rich fibrin

## Abstract

This study aimed to assess the effectiveness of photobiomodulation therapy (PBM) and leukocyte plasma-rich fibrin (L-PRF) in enhancing or accelerating osseointegration by promoting dental implant stability. The study included 15 patients lacking posterior mandibular teeth. Each participant received two implants in the mandibular edentulous area at the lower posterior region. The implants on the right side were categorized into three groups: Group A (650 nm laser), Group B (976 nm laser), and Group C with L-PRF (10 implants each). The left side served as the control. L-PRF was applied at the implant base before placement. Laser irradiation was performed on the buccal and lingual sides of the implants. Osseointegration was assessed using the AnyCheck system from Neo-Biotic Company. The Implant Stability Quotient (ISQ), ranging from 0-100, was measured post-operatively and after three months. There was a significant increase in stability after three months, particularly with 650 nm laser photobiomodulation followed by L-PRF application. The differences in ISQ among the three groups were statistically significant. After three months, implant stability was significantly higher both buccally and lingually in Group A, using powers of 75 mW and 100 mW, respectively, compared to the control (*P* <0.01). These findings indicate that PBM and L-PRF can significantly enhance osseointegration and improve dental implant stability in the posterior mandible. Patients and clinicians should consider the potential benefits of these interventions in implant dentistry, especially in cases involving the posterior mandible, where stability is crucial for successful implant outcomes.

## INTRODUCTION

Dental implant treatment has transformed oral rehabilitation in partially and fully edentulous patients [1]. Implant stability is one of the most crucial elements influencing the healing and successful osseointegration of dental implants [2]. The success of dental implants depends heavily on osseointegration, which is described as "the direct structural and functional between ordered, living bone and the surface of a load-carrying implant [3]. The bone, a vascularized connective tissue, can remodel in response to various influences and regenerate following injuries or pathological conditions. This regeneration encompasses complex intercellular and intracellular biological interactions, engaging different cell types and molecular signaling pathways [4,5]. Bone defects remain a significant health concern, particularly in elderly patients, contributing to substantial morbidity. In clinical applications, composites based on collagen/calcium phosphate have gained widespread use for bone repair due to their similarity to the extracellular matrix of the bone [6].

An adequate blood supply and angiogenesis are crucial prerequisites for successful bone regeneration. Angiogenesis, the formation of new blood vessels, is fundamental for achieving optimal regeneration outcomes [7]. It is worth noting that the remarkable feature of bone tissue is its capacity to heal without forming fibrous scars, setting it apart from many other tissues [8]. In medical treatments, photodynamic therapy (PDT) utilizes a photosensitizer activated by specific wavelength light, creating cytotoxic radicals that target tumor cells and induce inflammatory responses [9]. Leukocyte and platelet-rich fibrin (L-PRF) and low-level laser therapy (LLLT) are two new approaches for improving osseointegration around dental implants. L-PRF and LLLT are becoming increasingly used in dentistry today [10]. Photobiomodulation (PBM) is a non-invasive technique that can be strengthened to accelerate biological activities, including adenosine triphosphate (ATP) generation and DNA and RNA synthesis. Numerous studies have also demonstrated its importance for collagen formation, osteoblast proliferation and differentiation, bone repair, and rejuvenation-inducing mitosis [11,12]. Additionally, photobiomodulation shows promise in influencing gene regulation through epigenetic chromatin modifications. These modifications can occur independently of changes in the DNA sequence and are triggered by cellular responses to environmental alterations. Noncoding RNAs (ncRNAs) often serve as initiators in this process, identifying specific chromosomal regions requiring modification to influence gene expression. Histone modifications and DNA methylation, recognized as crucial epigenetic regulators, help maintain the stability of extensively modified chromatin.

Furthermore, tissue regeneration heavily relies on stem cells, which play a pivotal role in this process. Stem cells can be derived from embryonic or adult stem cells of postnatal origin [13]. These cells can differentiate into various cell types, making them essential contributors to tissue regeneration and repair.

L-PRF is a second-generation platelet concentrate that contains leukocytes and cytokines within a fibrin matrix [14]. In general, platelet-rich fibrin (PRF) is a simple, cost-effective, and minimally invasive technique to obtain a natural concentration of autologous growth factors. It is investigated in various medical fields for its potential to aid in regenerating tissues with low healing potential [15]. Dental implant stability is achieved through two phases: primary mechanical stability and secondary stability, which relies on the biological process of osteointegration [16]. Studies suggest that using PBM or L-PRF can significantly accelerate osteointegration, potentially leading to faster and more successful dental implant treatments. This is particularly relevant in a clinical setting where patients value shorter treatment durations and reduced post-operative discomfort [17].

Previous research has demonstrated the promising potential of PBM in promoting tissue healing, reducing post-operative pain, and stimulating bone regeneration. The field of PBM-induced biostimulation for bone tissue healing has rapidly expanded, with multiple studies showing promising outcomes [18]. Preclinical data in dentistry indicate that PBM positively affects bone healing and osseointegration, leading to its acceptance as a well-established adjuvant therapy to enhance osseointegration in cases of rehabilitation using implant-supported prostheses [19]. Various instruments and techniques have been developed to evaluate implant stability, including the insertion torque test, cutting torque resistance analysis, reverse torque test, mobility measurement test, and resonance frequency analysis. Although percussion and radiographic assessment are frequently used, especially in encapsulated situations, their results are not always accurate [20]. The Any Check device or Implant Stability Test (IST) represents a recent innovation in this field, offering a noninvasive, objective assessment of the alveolar bone-implant interface stiffness, thereby signaling implant failure risk [21]. This study aimed to investigate the clinical efficacy of PBM and L-PRF in enhancing dental implant stability. While previous research has individually explored PBM, L-PRF, and other osseointegration enhancement methods, our study uniquely focused on their application in the context of dental implant stability. By examining the effects of PBM and L-PRF on implant stability, this study aimed to reduce the osseointegration period, potentially leading to more efficient dental implant procedures and minimizing post-operative discomfort.

## MATERIAL AND METHODS

### Study design and participants

This study involved the placement of 30 dental implants in the posterior mandible of 15 patients. All implants, provided by Neo-Biotics, featured a sand-blasted and acid-etched surface. Patients were randomly divided into three groups, each consisting of five participants. Each patient received two implants, one on each side of the mandible. The left side acted as the control with normal loading and no additional treatment, while the right side was the study group.

**Group A:** In this group, five patients received PBM using a 650 nm laser on the right side. The laser power varied across patients (25 mW, 50 mW, 75 mW, 100 mW, 150 mW). The laser was applied for 40 seconds at two points (buccal and lingual sides) of each implant.

**Group B:** This group involved five patients with PBM using a 976 nm laser on the right side. The laser powers used were 0.05W, 0.1W, 0.15W, 0.2W, and 0.4W, with the same duration and application points as Group A.

**Group C:** The left side of each patient served as a control with standard implant procedures without adding L-PRF or PBM. On the right side, the L-PRF protocol was applied. This involved drawing blood into a 10-mL tube without anticoagulants and centrifuging it at 2700 rpm for 12 minutes, resulting in three layers: red blood cells, platelet-deprived plasma, and a fibrin gel rich in growth factors. The top plasma layer was removed, and the fibrin gel was extracted, shaped into a membrane, and placed into the osteotomy site before implant placement. This approach aimed to enhance osseointegration on the right side of the mandible.

### Implant stability measurement

Implant stability was assessed using the Any Check device or IST. This device taps the implant to measure the stiffness of the alveolar bone-implant interface, indicating the optimal time for prosthetic attachments.

### Inclusion and exclusion criteria

Participants aged 30 to 50 years, missing lower posterior teeth, and with edentulous regions for at least 6 months were included. Patients with significant bone loss, healing-impairing diseases (like diabetes and thyroid disorders), potential hormonal changes, or those undergoing radiation/chemotherapy were excluded.

### Surgical procedure

All surgeries were performed under local anesthesia (2% lidocaine with 1:100,000 epinephrine, Novocol Pharmaceutical). The lower posterior areas of both the left and right mandibles were prepared with a horizontal mid-crestal incision using a #15 Bard-Parker blade. The incisions were made through the connected gingiva, positioned lingually to the crest of the alveolar ridge, approximately 3–4 mm from the crest. Following the incision, a mucoperiosteal flap was bluntly dissected using a periosteal elevator, allowing exposure on both the buccal and lingual sides of the alveolar ridge. Osteotomy procedures began with a pilot hole of 2.0 mm diameter, drilled using a starting drill. This was followed by additional drills, operating at the manufacturer-recommended speed of 800 rpm, to prepare the site for implant placement.

**Figure 1:**
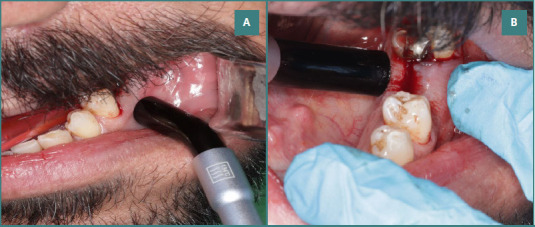
Laser irradiation points during dental implant procedure. A, buccal side; B, lingual side

For groups A and B, we utilized a red diode laser (LX 16 WOOD PEACKER from Guilin Guangxi) set at 650 nm and 976 nm. The laser application involved a bio-modulating handpiece with variable output powers, a handpiece diameter of 8mm, and a spot area of 0.5024 cm^2^. Each laser point was applied for 40 seconds, targeting two points – the buccal and lingual sides of the implant site. This is illustrated in Figure 1 (AB). The diode laser, in contact mode, was applied to the peri-implant soft tissue immediately after surgery and on subsequent days (3, 5, 7, 9, 11, 13, 15, 17, and 19 days post-surgery) as part of the treatment protocol for these groups.

### Statistical analysis

Statistical analyses were performed using SPSS software (version 20). A repeated measures ANOVA was used to compare experimental groups to the control group, with data presented as mean ± standard deviation (SD). Significant differences between group means were determined using the Least Significant Difference (LSD) test.

## RESULTS

### Implant Stability Quotient (ISQ) measurements

Implant Stability Quotient (ISQ) measurements were conducted bucco-lingually around the implants using the Any Check device. This device assesses the stiffness of the alveolar bone-implant interface through a tapping motion. Measurements were taken immediately after dental implant insertion and at the 3-month mark. A gingival former, 4 mm in height, was placed and tightened to 15 Ncm on the implant per manufacturer instructions. The Any Check device was then used to measure stability, ensuring the tapping rod made slight contact with the upper part of the healing abutment at an angle between 0 and 30 degrees. The standard ISQ range is 1 to 100, with 100 indicating the highest implant stability.

### Group A: 650 nm laser application

**Figure 2:**
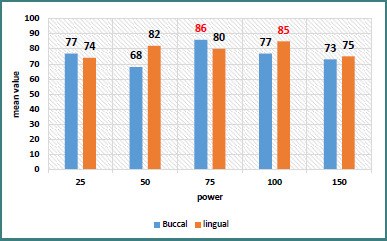
Comparison of ISQ mean values for buccal and lingual directions at 90 days across different laser powers (650 nm laser application)

Buccal side: At 90 days, the 75-mW power setting showed the best ISQ results (86 ± 2.4), indicating optimal outcomes at this power level (Table 1, Figure 2).

**Table 1:** ISQ measurements on the buccal side between baseline and three months in group A

Power	1 Day Buccal (Mean ± SD)	90 Day Buccal (Mean ± SD)	*P* value
25	67 ± 3.2	77 ± 2.7	0.05
Control	65 ± 3.1	68 ± 2.4	NS
*P* value	NS	0.05	
50	59 ± 2.9	68 ± 2.9	0.05
Control	62 ± 2.3	68 ± 2.5	NS
*P* value	NS	NS	
75	69 ± 3.4	86 ± 2.4	0.02
Control	68 ± 2.8	72 ± 2.5	NS
*P* value	NS	0.05	
100	64 ± 3.1	77 ± 2.4	0.05
Control	66 ± 3.5	71 ± 2.7	NS
*P* value	NS	NS	
150	62 ± 2.5	73 ± 2.3	0.05
Control	59 ± 2.4	63 ± 2.6	NS
*P* value	NS	0.05	

NS, not significant

Lingual side: The 100-mW power setting demonstrated superior results at baseline and 3 months, with the highest mean ISQ (85) and a statistically significant difference at 3 months, indicating differences between the groups (Table 2, Figure 2).

**Table 2:** ISQ measurements on the lingual side between baseline and three months in group A

Power	1 Day Lingual (Mean ± SD)	90 Day Lingual (Mean ± SD)	*P* value (1 Day)	*P* value (90 Day)
25	60 ± 2.2	74 ± 1.7	NS	0.05
Control	59 ± 2.1	64 ± 1.4	NS	NS
*P* value	NS	0.05		
50	70 ± 2.9	82 ± 1.9	NS	0.05
Control	66 ± 2.2	72 ± 1.5	NS	0.05
*P* value	NS	0.05		
75	60 ± 2.4	80 ± 1.4	NS	0.01
Control	66 ± 2.8	70 ± 1.5	NS	0.05
*P* value	NS	0.05		
100	70 ± 2.1	85 ± 1.4	NS	0.01
Control	74 ± 2.5	76 ± 1.7	NS	NS
*P* value	NS	0.05		
150	60 ± 2.8	75 ± 1.3	NS	0.01
Control	63 ± 2.9	67 ± 1.6	NS	NS
*P* value	NS	0.05		

NS, Non-significant.

*P* value between all tested groups (powers) at 1 day: non-significant.

*P* value between all tested groups (powers) at 90 days: significant at 0.05.

### Group B: 976 nm laser application

**Figure 3:**
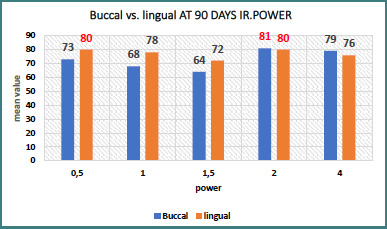
Comparison of ISQ mean values for buccal and lingual directions at 90 days across different laser powers (976 nm laser application)

Buccal side: At 90 days, the 0.2 W power setting yielded the highest mean value (81), suggesting favorable implant stability (Table 3, Figure 3).

**Table 3:** ISQ measurements on the buccal side between baseline and three months in group B

Power	1 Day Buccal (Mean ± SD)	90 Day Buccal (Mean ± SD)	*P* value
50	62 ± 2.8	73 ± 2.3	0.05
Control	61 ± 2.3	63 ± 2.6	NS
*P* value	NS	0.05	
100	64 ± 2.2	68 ± 3.1	NS
Control	66 ± 2.1	69 ± 3.2	NS
*P* value	NS	NS	
150	59 ± 2.9	64 ± 2.8	NS
Control	62 ± 2.7	68 ± 2.3	NS
*P* value	NS	NS	
200	67 ± 3.1	81 ± 2.3	0.05
Control	70 ± 3.3	75 ± 2.8	NS
*P* value	NS	0.04	
400	62 ± 2.4	79 ± 2.6	0.05
Control	59 ± 2.1	64 ± 2.9	NS
*P* value	NS	0.04	

Lingual side: The highest mean values were observed at 0.2 W and 0.05 W, with a mean of 80, although the *P* value was not significant (Table 4, Figure 3).

**Table 4:** ISQ measurements on the lingual side between baseline and three months in group B

Power	1 Day Lingual (Mean ± SD)	90 Day Lingual (Mean ± SD)	*P* value
50	66 ± 1.8	80 ± 3.3	0.01
Control	65 ± 1.3	68 ± 3.6	NS
*P* value	NS		
100	70 ± 1.1	78 ± 3.1	0.05
Control	71 ± 1.1	73 ± 3.3	NS
*P* value	NS		
150	63 ± 1.9	72 ± 3.2	0.01
Control	66 ± 1.7	68 ± 3.3	NS
*P* value	NS		
200	63 ± 3.1	80 ± 3.3	0.01
Control	62 ± 3.3	65 ± 3.2	NS
*P* value	NS		
400	55 ± 1.4	76 ± 3.6	0.01
Control	60 ± 1.1	60 ± 1.2	NS
*P* value	NS	0.04	

NS, not significant

### Group C: L-PRF application

Buccal side: The highest mean value was 78 ± 3.1 for 1-growth factor concentration (GF), while for 5-GF it was 75 ± 2.3.

**Figure 4:**
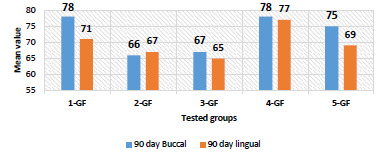
Comparison of ISQ mean values for buccal and lingual directions at 90 days across different growth factor concentrations

Lingual side: The highest mean value was 77 ± 1.9 for 4-GF (Table 5, Figure 4).

**Table 5:** Comparison of ISQ measurements on the buccal and lingual sides between baseline and three months for GF groups

GF Group	One Day Buccal (Mean ± SD)	90 Day Buccal (Mean ± SD)	*P* value (Buccal)	One Day Lingual (Mean ± SD)	90 Day Lingual (Mean ± SD)	*P* value (Lingual)	*P* value (B vs L at 90 Day)
Control	65 ± 3.1	67 ± 2.9	NS	62 ± 2.1	65 ± 2.1	NS	NS
1-GF	68 ± 2.6	78 ± 3.1	0.05	64 ± 1.9	71 ± 2.3	0.05	0.05
Control	61 ± 1.9	66 ± 2.7	0.05	59 ± 2.3	67 ± 2.8	0.05	NS
2-GF	62 ± 2.8	66 ± 2.1	NS	59 ± 2.3	67 ± 2.8	0.05	NS
Control	72 ± 2.6	75 ± 2.1	NS	67 ± 2.1	70 ± 2.1	NS	NS
3-GF	60 ± 2.4	67 ± 2.9	0.05	58 ± 1.7	65 ± 2.8	0.05	NS
Control	68 ± 2.6	72 ± 3.1	NS	67 ± 1.9	69 ± 2.1	NS	NS
4-GF	70 ± 2.8	78 ± 2.8	0.04	73 ± 2.3	77 ± 1.9	NS	NS
Control	71 ± 2.1	74 ± 2.9	NS	68 ± 2.7	73 ± 1.9	NS	NS
5-GF	63 ± 2.5	75 ± 2.3	0.04	61 ± 2.1	69 ± 2.3	0.05	0.05

### Comparative analysis

Our comparative analysis of the ISQ across the 650 nm, 976 nm, and L-PRF (GF) groups showed significant differences in the bucco-lingual direction from implant placement to three months post-operation. The highest efficacy on the buccal side was seen with the 75-mW power setting at 650 nm, yielding an average ISQ value of 86, followed closely by the 0.2 W setting at 976 nm with an average value of 81. On the lingual side, the 100-mW setting at 650 nm led with an average ISQ value of 85, while the 976 nm group at 0.05 W showed a high mean value of 80. The L-PRF group had the least impact on implant stability, with mean ISQ values of 78 buccally and 77 lingually (Table 6).

**Table 6:** Comparison of ISQ measurements on the buccal and lingual sides between baseline and three months across different groups

Power	75 Visible light	0.2 IR	GF ANY CHECK	*P* VALUE
Buccal	A 86 ± 2.4	B 81 ± 2.3	C 1-GF = 78 ± 3.1 4-GF = 78 ± 2.8	0.01
Power	100 Visible light	IR	GF ANY CHECK	
Lingual	A 85 ± 1.4	B 50 = 80 ± 3.3 200 = 80 ± 3.1	C 4-GF = 77 ± 1.9	0.01
*P* VALUE	NS	NS	NS	

## DISCUSSION

The primary therapeutic effects of PBM therapy, as observed in our study, include tissue bio-stimulation, acceleration of the healing process, promotion of bone regeneration, and reduction of pain [22]. Despite being intricate, the PBM process primarily depends on activating cytochrome c-oxidase at the mitochondrial electron transport chain to absorb specific visible red and near-infrared wavelengths [23,24]. The mitochondria may increase ATP production, RNA and DNA, and protein synthesis by absorbing PBM [25,26]. Various lasers and biomaterials have been used for such studies [27]. Each laser has a precise property because it emits different wavelengths, promoting a different tissue interaction [28]. Irradiation parameters that ensure safe clinical use of such dual wavelength laser should be defined before any in vivo application [29].

Our study found the most effective power settings for implant stability on both the buccal and lingual sides to be 75 mW and 100 mW at 650 nm, respectively. This is likely due to the higher absorption rate in the visible region. In contrast, the optimal power settings for 976 nm were 0.2 W and 0.05 W. The lower scattering and absorption by tissue chromophores at near-infrared wavelengths allow for more efficient energy absorption by osteoblasts, the cells responsible for bone formation [30,31]. As indicated in Table 6, the most effective method for accelerating osseointegration on the buccal side was the application of L-PRF. Choukroun's PRF is a matrix that can act as a resorbable membrane and entrap cells and cytokines that are then released after a brief time [32]. The positive effects of 976 nm diode laser irradiation on alveolar bone sockets, observed in both superficial and deeper tissues, align with the findings of Park *et al*. [33]. This suggests that PBM at this wavelength can effectively stimulate bone healing and regeneration. Analyses of gene expression and observations showed that the 980 nm diode laser irradiations speed up bone regeneration and reduce the number of inflammatory cells while increasing the number of fibroblasts and osteoblasts [33]. The effects of PBM are dose-dependent, as described by the Arndt-Schultz curve, with higher energy doses generally leading to greater effects. The optimal PBM spectrum for tissue penetration lies within the optical window of 600 to 1100 nm, resulting in a stronger light-to-cell interaction and enhanced therapeutic response [34]. In the laser group, secondary stability was significantly higher at the 3-month evaluation compared to the baseline and the control group. This finding aligns with other studies demonstrating that PBM accelerates the healing process after injury by promoting new bone growth [35,36]. However, some studies reported no significant effects of PBM on implant stability in healed sites [37-39]. This discrepancy could be due to the chosen wavelength, very low or high dose, irradiation time, number of therapy sessions and the time between treatments, spot size, inadequate understanding of laser-tissue interaction, and inaccurate diagnosis [40]. In addition to enhancing implant stability, PBM can hasten healing close to the surgery site by enhancing ATP production and angiogenesis, reducing inflammation, and enhancing osteoblast proliferation [34]. High initial stability, implant shape, and good bone quality are implantology characteristics that have a greater impact on the quality of the implant-bone interface than any further therapeutic effort [41]. The findings of Pirpir *et al*. [42] and Incü *et al*. [43] align with our results, showing a significant increase in ISQ in the platelet concentrates group. Conversely, a systematic review by Fujioka-Kobayashi *et al*. suggested a lesser impact of PRF on implant therapy and bone regeneration [44]. They concluded that PRF may have a limited effect on bone regeneration, sinus elevation, and implant therapy outcomes. Despite this discrepancy, our study reaffirms the positive impact of PBM energy on bone repair. Additionally, our findings support the hypothesis that L-PRF possesses characteristics that enable the sustained release of growth factors, contributing to improved tissue regeneration and implant stability.

The study explored a relatively novel approach by combining PBM and L-PRF to enhance dental implant stability. This unique approach contributes to the novelty of the study and may provide valuable insights. The study addresses a clinically relevant question by investigating whether PBM and L-PRF can reduce the time required for osseointegration. Shorter osseointegration periods can lead to improved patient experiences and reduced treatment durations. The study includes a control group, essential for making meaningful comparisons and assessing the specific effects of PBM and L-PRF on implant stability. The use of ISQ measurements from the Anycheck device provides quantitative data, enhancing the objectivity of the results. The study includes 15 patients, which, while relatively small, is still a reasonable sample size for a clinical study of this nature.

One of the limitations of this study is the small sample size, which may reduce the generalizability of the findings. Larger sample sizes are typically preferred for increased statistical power. The assessment of implant stability was conducted only up to the third postoperative month. A longer-term follow-up would provide a more comprehensive understanding of the durability of the effects observed. In addition, we utilized five different laser power settings without a detailed rationale for selecting these specific powers. A more comprehensive exploration of different power settings could yield valuable insights. Finally, the study was conducted at a single center, which may limit the diversity of patient populations and treatment protocols considered. Future research should include longer-term follow-up assessments to determine the sustainability of the observed effects. This would provide a more comprehensive evaluation of the clinical significance of PBM and L-PRF in implant dentistry.

## CONCLUSION

Innovative methods such as PBM and L-PRF significantly improved the osseointegration of dental implants. These advanced techniques hold the potential to increase the success rates of dental implants. Our study concludes that PBM has a greater effect on osseointegration compared to L-PRF. However, the combined use of L-PRF and PBM may further enhance dental implant osseointegration compared to using PBM alone.

## Personal thanks

The authors thank the Woodpecker company for supporting the sensor equipment.
